# Functional respiratory imaging of airways in ventilated ARDS patients: revealing the regional relation between PEEP-induced airway opening and airway dilatation

**DOI:** 10.1186/cc14325

**Published:** 2015-03-16

**Authors:** T Schepens, C Vanholsbeke, W Vos, J De Backer, P Parizel, PG Jorens

**Affiliations:** 1 Antwerp University Hospital, Edegem, Belgium; 2 FLUIDDA, Kontich, Belgium

## Introduction

ARDS has a wide variability of lung morphological characteristics. Both alveolar collapse and airway narrowing or closing are present, often heterogeneously. Despite advances in ARDS imaging, we have thus far been unable to distinguish regional airway opening from airway dilatation in PEEP-induced lung recruitment. We demonstrate the technique of functional respiratory imaging (FRI) to differentiate these two entities.

## Methods

Six patients with early-stage ARDS were included in this prospective single-centre cohort trial. The lower infliction point on a pressure-volume curve was considered as the clinically acceptable minimal PEEP value. Subsequently, four distinct PEEP levels were chosen to perform CT scans: at 20 cmH_2_O; median value between 1 and 3; clinically acceptable minimal; and 0 cmH_2_O. FRI methods as described by De Backer et al. [1] were used to evaluate airway opening and airway dilatation.

## Results

**Figure 1:**
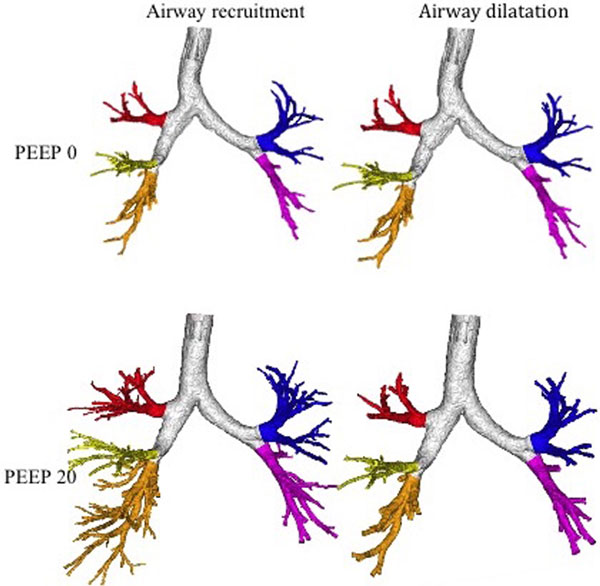
**Bronchodilation and bronchial recruitment split per lobe for one patient**.

Airway stretching (that is, bronchodilatation) could be quantified and distinguished from airway recruitment with this technique. Higher PEEP pressures not only recruit, but also expand the bronchi. The ratio of dilation/recruitment of bronchi was higher in the upper lobes than in the lower lobes, as illustrated in Figure 1. We were able to phenotype each patient, allowing a prediction on when an increase in PEEP further recruits atelectasis/bronchi or distends certain airway regions.

## Conclusion

The novel technique of FRI can be used to visualise the airway structures in ARDS and distinguish airway stretching from airway recruitment. This pilot study shows that, in ARDS, the upper lung regions are subject to airway dilation, whereas the lower (atelectatic) lung lobes have more airway opening with higher PEEP levels.
